# An exploration of alcohol use amongst undergraduate female psychology students at a South African university

**DOI:** 10.4102/sajpsychiatry.v23i0.1022

**Published:** 2017-04-10

**Authors:** Indiran Govender, Kathryn Nel, Xolile M. Sibuyi

**Affiliations:** 1 Department of Family Medicine and Primary Health Care, Sefako Makgatho Health Sciences University, South Africa; 2 Department of Psychology, University of Limpopo, South Africa; 3 Elim Hospital, South Africa

## Abstract

**Background:**

Alcohol use amongst tertiary education students, particularly female undergraduates, is increasing. Heavy alcohol use by tertiary students leads to a variety of alcohol-related problems such as damage to property, poor academic performance, problematic peer relationships, high dropout rates, unprotected sexual activity, physical injuries, date rape and suicide. Abuse of alcohol is attributed to curiosity and experimentation, peer pressure, low self-esteem, enjoyment, parental modelling, socio-cultural influences, stress and life events, self-medication and concerns about weight and appearance.

Our study explores alcohol use and the reasons behind it amongst undergraduate female psychology students at the University of Limpopo. The findings will be important, as these students represent many future psychologists who are going to advise others on harms related to alcohol use.

**Methods:**

This was a descriptive survey, and the qualitative results are presented. The sample consists of 700 undergraduate female psychology students. A self-administered questionnaire included five open-ended questions which elicited the thoughts and experiences of these students about alcohol use. Responses to these questions were analysed using thematic content analysis.

**Results:**

The themes that arose were as follows: fun and enjoyment, socio-cultural influences, alcohol use leads to negative behaviour(s), peer influence, destress, concerns about weight and appearance, abstinence from alcohol and it improves self-esteem.

**Conclusion:**

The themes were reasons that female students gave for consuming alcohol. The majority of participants reported responsible drinking behaviour, but a notable proportion of female students’ drinking behaviours (across all year levels) are cause for concern in terms of negative impact at both social and academic levels.

## Introduction

Household surveys in South Africa found nearly 50% of males and 20% of females consumed alcohol regularly – and this is probably an underestimate.^1^ In South Africa, one-third of those who consume alcohol are reported to have risky drinking behaviours over weekends, such as binge drinking.^2^

Alcohol use amongst tertiary education students, particularly female undergraduates, is an increasing problem. Heavy alcohol use by tertiary students leads to a variety of alcohol-related problems, such as property damage, poor academic performance, problematic peer relationships, high dropout rates, unprotected sexual activity, physical injuries, date rape and suicide.^1,3^ Tertiary education students in America and England consume more alcohol than those of similar age who do not attend university.^4^ University students see the heavy use of alcohol as normal and as something that is expected of them.^3^

University students, especially undergraduates, experiment with alcohol, which can lead to physical, social, emotional and academic problems.^1,5^ Hence, alcohol use by female university students can be harmful to the progress of their tertiary education. As undergraduate students are away from parental or caregiver supervision, they are likely to have an unrealistic sense of independence and may misuse alcohol.^6^

A tertiary institution in South Africa reported that males and females have similar drinking patterns in terms of when they drink, how much they drink and the types of alcohol they drink.^1^ Some students considered excessive drinking by both males and females as problematic.^1^ Females have a poor physiological tolerance for alcohol, which may result in long-term social or psychological problems.^7^

The South African Demographic and Health Survey found that 45% of males and one-fifth of females consume alcohol every week.^8^ Abuse of alcohol by female students is attributed to curiosity and experimentation, peer pressure, low self-esteem, enjoyment, parental modelling, socio-cultural influences, stress and life events, self-medication and concerns about weight and appearance.^1,9,10,11,12,13^

There is a scarcity of research on alcohol use amongst female university students. Our survey aims to fill this gap by investigating alcohol use amongst undergraduate female psychology students registered at the University of Limpopo. This exploration is important, as these students represent many future psychologists in South Africa who are going to advise other people on harms related to alcohol use, as well as assist people who have problematic drinking habits.

## Methods

This was a descriptive survey and the qualitative results are presented. The sample consists of 700 undergraduate female psychology students. A self-administered questionnaire included five open-ended questions which elicited their thoughts and experiences about alcohol use. These questions were adapted from a questionnaire used in a previous study about alcohol use amongst male and female students undertaken at the university.^1^ The questions were modified to make them relevant to female undergraduate students only and were piloted with 10 such students to determine face validity.^14^ As the respondents answered the questions appropriately, the questions were subjectively determined to measure what they were designed to measure.

In terms of ensuring the credibility of this study, the researchers adopted well-recognised research methods. To ensure confirmability, the researchers were aware of their beliefs and assumptions and bracketed them (put to one side)^14^ during the research process, as well as minimising any potential effect through reflection and discussion. Dependability was ensured by using a research method that was replicable. Overall validity was addressed by recording the number of participants and ensuring that data were interpreted in the same way. Participant bias^14^ was inevitable, as only females were included in the study. Administrative bias^14^ was avoided as researchers gave the same instructions to participants.

Questionnaires were distributed to all students, and all were administered in English, the language of teaching and training at this university. Responses to the questions were analysed using thematic content analysis.^14^

The five questions posed were as follows:

1. Do you or your fellow female students drink alcohol at social occasions like the freshers’ ball? If so, why?
2. Do you or your fellow female students drink alcohol when you or they are alone? If so, why?
3. Why do you or other female students consume alcohol?
4. What are your thoughts about drinking alcohol?
5. Is there anything else you would like to say about female students drinking alcohol?

### Setting of the study

The University of Limpopo (Turfloop campus), which is approximately 30 km east of Polokwane in Limpopo Province, was the setting, and the sample was registered female students in the first-, second- and third-year psychology classes.

### Data collection

Permission was obtained from the psychology undergraduate coordinators to hand out questionnaires in class. The students were asked to return the completed questionnaires to a marked box in the Department of Psychology. It took respondents 15–25 minutes to complete the questionnaires.

### Analysis of qualitative data

Data from the five open-ended questions were analysed using thematic content analysis, which is a descriptive presentation of qualitative data and a conventional practice in qualitative research. This involves searching through data to identify any recurrent patterns or themes. A theme is a cluster of linked categories conveying similar meanings and usually emerges through the inductive analytical process which characterises thematic content analysis.^14^ The following steps were followed to analyse the data: firstly, immersion within and familiarisation with the data so that the researcher gets to know the data intimately, the data were put into major categories and then minor categories, and the categories were grouped into themes which reflect the transcribed data. The researcher had to go through each of the steps again, reflecting on the process to ensure objectivity.^15^

## Ethical considerations

Informed consent was obtained from all participants. Anonymity and confidentiality were ensured throughout the research. Ethical clearance was obtained from the University of Limpopo’s research and ethics committee (Turfloop Ethics Committee TREC/FHM/42/2013: PG).

## Results

There were 130 completed questionnaires, which represented a response rate of 19%. Of these, 48 (37%) were from first-year students, 40 (31%) from second-year students and 42 (32%) from third-year students. Seventy-three (56.2%) of the students were aged between 18 and 21 years, and 115 (88.5%) were Christians (Table 1).

**TABLE 1:** Characteristics of the participants.

Characteristics	Frequency	%
**Age (years)**		
18–21	73	56.2
22–35	57	43.8
**Religion**		
Christian	115	88.5
Other religion	15	11.5
**Year of study**		
First	48	37.0
Second	40	31.0
Third	42	32.0
**Total**	**130**	**100.0**

### Emerging themes

The themes that emerged from the responses to the open-ended questions are summarised in Table 2, together with an explanation of the themes and associated excerpts from the questionnaires.

**TABLE 2:** Themes.

Theme	Explanation	Excerpts
Curiosity and experimentation	Participants drank alcohol, especially at parties, to experience the feeling of being drunk. They were experimenting with alcohol.	‘I drink at parties so I can know how it feels to be drunk and carefree like my friends.’
Fun and enjoyment	52 participants spoke about drinking alcohol to have fun.	‘it makes you fit in with my friends and have fun.’ ‘I feel great, I’m hyperactive, it is fun.’
Peer pressure or influence	22 participants talked about drinking alcohol to fit in with friends	‘I want to fit in the standard of my friends.’ ‘I keep drinking to keep up with my friends, they push me.’
Self-esteem	Some students do not have self-confidence and use alcohol to reduce their inhibitions – 30 participants found that drinking alcohol boosts their self-confidence or self-esteem.	‘I drink at social functions because I want to fit in the standard of my friends because I’m shy.’ ‘I drink alcohol so I feel better about myself, otherwise I feel all my friends are better than me.’
Socio-cultural influences	Students entering university enter a new culture and society. This influences their behaviour, including alcohol use.	‘I keep drinking to keep up with my friends, they push me.’ ‘I drink alcohol so I fit in with other students in the university.’
Reduces anxiety and stress	22 participants stated that alcohol makes them relaxed and destresses.	‘when I’m bored and no one cares about me, I drink alone to relax.’ ‘I feel good because it also acts as a relief from stress and I enjoy when I’m drunk.’ ‘I feel happy, great and good when I drink.’
Self-medication	Some female students used alcohol to self-medicate to improve their mood and to feel better.	‘I destress and drown my sorrows in alcohol.’
Lack of support	A proportion of female students reported that they used alcohol when they have difficulties in establishing or maintaining supportive relationships.	‘When I’m bored and no one cares about me, I drink alone to relax.’
Concerns about weight and appearance	Students stated that when they drink alcohol they do not think about the concerns they usually have about their weight and appearance. Alcohol removes this burden from their minds.	‘At parties if I am drunk I don’t care that I am fat, I feel free.’
Abstinence	Some of the participants abstain totally from alcohol and would prefer it if all people abstained – seven participants spoke about abstaining from alcohol use.	‘People should abstain from drinking alcohol at all.’ ‘Many don’t drink at all.’ ‘Many students don’t drink at all at social functions.’
Negative behaviour(s) – alcohol is unhealthy and leads to bad behaviour	Respondents indicated that drinking alcohol is unhealthy and it leads to bad behaviour: 34 participants were concerned about the bad behaviour female students exhibit when they drink alcohol.	‘It’s bad for females’ health they should stop.’ ‘Drinking alcohol is not a good thing, because when you are drunk you tend to do things that are out of order and bad.’ ‘drinking is not good for ladies because most of them get raped because of being drunk while some are involved in fights.’

## Discussion

The themes that emerged are explored in relation to understanding what these female students think about drinking alcohol.

### Causes of alcohol abuse

The following factors related to the use of alcohol were reinforced by the emerging themes.

#### Curiosity and experimentation

‘I drink at parties so I can know how it feels to be drunk and carefree like my friends.’

Many people sample alcohol when they are young and at home during special occasions and some religious festivals. Out of curiosity, they taste alcohol in its various forms, in various combinations and often in increasing quantities. When overindulgence occurs, it represents the youngsters’ attempt to define his or her capacity, as well as to experience how it feels to be drunk. Unlike adult abusers, who deliberately become intoxicated, inexperienced drinkers usually do not intend to get drunk but may become so when experimenting with alcohol. Because of peer pressure and activities such as parties and going to nightclubs, experimentation ends and drinking alcohol becomes the norm. This curiosity may extend to alcohol abuse later on in life.^9^ Although this experimentation seems harmless at first, there are major consequences such as high dropout rates amongst students who become heavy alcohol users while studying, as well as reported and unreported acts of sexual violence.^1,3^

#### Fun and enjoyment

‘it makes you fit in with my friends and have fun.’

Some female students have the belief that a social activity cannot be enjoyed unless it includes the consumption of alcohol. Many of them have the mentality that they will not enjoy a party, a movie or school trip unless they are feeling the effects of alcohol. They are susceptible to this point of view because of peer pressure and media advertising, plus their innate rebelliousness against society. Adults drink alcohol as often they feel that it adds enjoyment to a meal or social occasion; when done in moderation, this is true, but over-indulgence (which is often ignored) can sometimes have fatal consequences (for instance, drinking and driving, marital disputes ending in violence).^9^

#### Peer pressure or influence

‘I want to fit in the standard of my friends.’

Peer pressure may be more strongly associated with drinking for females than males. High school girls who report high levels of peer pressure to drink are twice as likely to use alcohol as those reporting less peer pressure.^1,10^ This relationship between peer pressure and alcohol consumption was not found for boys. When several of a young female’s closest friends smoke or drink, they are more than seven times more likely to drink alcohol (boys who have several close friends who smoke or drink are only three times more likely to drink alcohol).^1,10^ The change from adolescence to young adult leads to a greater vulnerability to the use and abuse of alcohol, particularly amongst those who study at tertiary institutuons.^10^

#### Low self-esteem

‘I drink alcohol so I feel better about myself, otherwise I feel all my friends are better than me.’

Female university students with low self-esteem or low self-confidence are twice as likely as those with higher self-esteem (self-confidence) to abuse alcohol. One study found that girls who have low self-esteem at the age of 12 were nearly 2.5 times more likely to engage in heavy alcohol use at age 18 than those with higher self-esteem.^13^

#### Socio-cultural influences

‘I keep drinking to keep up with my friends, they push me.’

Several factors contribute to an individual’s decision to drink or not drink on any given occasion. The most obvious are those in the immediate environment, such as the nature of the occasion, an individual’s personal interest, and physical and mental state, and the degree of peer pressure. There are also socio-cultural influences including historical and contemporary factors which contribute to an individual’s attitudes, beliefs and values regarding the use of alcohol. These may include religious, ethnic and family customs that are mediated by environmental factors in tertiary environments.^16^ When an individual with a certain set of beliefs and expectations about drinking comes into contact with individuals who have different definitions of alcohol use, the individual may be put into a situation which demands social drinking at a level that he or she previously would have considered as alcohol misuse.^12^ These drinking patterns are motivated by peer pressure and trying to fit in or have an acceptable social identity.^12^

#### Reduces anxiety and stress: Alcohol used to destress

‘I feel good because it also acts as a relief from stress and I enjoy when I’m drunk.’

Many young women who are alcoholics give premenstrual depression as a reason for their drinking. Furthermore, an increasing number of women report an increased intake of alcohol during their menses. Adolescents receiving treatment for alcohol or drug abuse have pointed out undesirable life events as the cause of their drinking behaviour.^9^ It was noted in the research that there was a relationship between heavy drinking and financial, academic and family stressors amongst young adults. High rates of substance abuse are associated with negative life experiences or events such as parents getting divorced, conflicts between parents and in females the onset of menstruation. Furthermore, it is reported that drug use is related to experiencing stressful life events. Individuals who have not learned coping strategies to deal with stress may resort to heavy consumption of alcohol.^12^

#### Self-medication

‘I destress and drown my sorrows in alcohol.’

Females who believe that drinking alcohol alleviates boredom or helps them to deal with sadness or depression are reported to use more alcohol than those who do not. Females appear to be attuned to the self-medicating powers of alcohol as early as junior school. Females are more likely than males to believe that the positive effect of alcohol is its ability to alleviate bad moods or feelings. It is also reported that female university students who drink heavily are more likely to have a pattern of hazardous alcohol consumption (HAC) than males. For instance, in one study of college students in Ireland, females had a 67.3% HAC compared with males at 65.2%, and 57.4% of the females had the same HAC threshold as males. These students also reported smoking and illegal drug use.^13^

In his study of undergraduate medical students, Govender^1^ also noted that alcohol is sometimes used by students to help them cope with their personal problems, as they feel that they are able to express themselves more openly under its influence. Female students are more likely to self-medicate with alcohol.

#### Lack of support

‘When I’m bored and no one cares about me, I drink alone to relax.’

Young female students who have a hard time building and maintaining healthy relationships might turn to alcohol. An adolescent with absent parents, few friends and trouble meeting new people may feel lonely and become depressed. Lack of emotional support and low self-esteem are closely linked. Many female students develop low self-esteem.^12^ This is supported by research which indicates that they usually score higher on stress-related tests than males and are also likely to self-medicate to relieve anxiety and stress by using alcohol. Parent drinking behaviour and favourable attitudes about drinking have been positively associated with adolescents initiating and continuing drinking. Lack of parental support, monitoring and communication have been seen to predict adolescent drinking and alcohol-related problems.^17^

#### Concerns about weight and appearance

‘At parties if I am drunk I don’t care that I am fat, I feel free.’

Females who perceive themselves as being overweight, who are actively trying to lose weight, or who engage in unhealthy dieting behaviours drink more alcohol than females with healthier weight-related attitudes and behaviours.^13^ As more females are becoming concerned about their weight because of media portrayals of very thin women, this issue is becoming very problematic.^1^

The opinions and experiences given as reasons why these students consume alcohol, as described above, are similar to other students’ experiences reported globally.^12,13^

### Other thoughts about female students drinking alcohol

#### Abstinence from alcohol

‘Many don’t drink at all.’

Some of the participants totally abstain from alcohol, and they report that other female students also abstain. They feel that all people (not only female students) who are drinking alcohol should abstain completely. This theme points to strong self-efficacy amongst these students. Research has shown that this ability is a strong determinant in preventing problematic alcohol consumption amongst undergraduate students, and it also indicates the students’ ability to overcome strong external influences (such as peer pressure) at university.^18^

#### Negative behaviour(s)

‘Drinking is not good for ladies because most them get raped because of being drunk while some are involved in fights.’

Participants reported that alcohol consumption leads to bad behaviours. They were concerned about the bad and sometimes risky behaviours of female students when they drink alcohol. Students need to be aware of the potential dangers associated with negative behaviours while drunk.

Drinking of alcohol amongst female university students is associated with a variety of negative consequences. These include fatal and nonfatal injuries; alcohol poisoning; blackouts; academic failure; violence, including rape and assault; unintended pregnancy; sexually transmitted diseases, including AIDS; property damage; and vocational and criminal consequences that could negatively influence their future job prospects.^18^

Some students show insight into the negative influences which alcohol could have on their life, whereas others display self-resilience with their view on abstinence in spite of the pressures of university life. This shows their self-efficacy, which can be used for positive peer influence.

## Conclusion

The themes that arose in this study are in keeping with those found in existing literature on the reasons that female students consume alcohol. These were curiosity and experimentation, peer pressure or influence, low self-esteem, fun and enjoyment, socio-cultural influences, stress and life events, self-medication, lack of support and concerns about weight and appearance. Students also expressed the views that alcohol leads to negative behaviour and female students should abstain from alcohol use.

Future research should be carried out into alcohol use amongst both genders registered at different year levels, with a qualitative study investigating the experience of alcohol use amongst both sexes. University management needs to take heed of the views and experiences of students in this research, and measures should be considered to assist students in improving their self-efficacy. Other interventions that may be considered by the university include having alcohol awareness campaigns during all levels of study, but particularly during first-year orientation, where students will first be introduced into a new society and culture. The University of Limpopo may also consider distributing pamphlets at outlets such as the clinic and library informing students of problems associated with alcohol use.

## Limitations

This was a qualitative study amongst undergraduate female psychology students at one university, and as such, the results are not generalisable to all university students.
